# National COVID-19 lockdown exit strategies need to pay more attention to community engagement and workplace safety

**DOI:** 10.7189/jogh.10.020323

**Published:** 2020-12

**Authors:** Kathrin Cresswell, Sangeeta Dhami, Aziz Sheikh

**Affiliations:** 1 Usher Institute of Population Health Sciences and Informatics, The University of Edinburgh, UK; 2 GP Locum, Edinburgh, UK

The emergence of SARS-CoV-2 in December 2019 has resulted in the unprecedented lockdown of over 4.5 billion people globally in an attempt to reduce virus transmission, infection and death rates, and to protect health care systems [1]. However, with concerns mounting regarding the unintended health (eg, through disruption to immunisation programmes), societal (eg, mental health) and economic effects of lockdown, there is mounting pressure in many countries to relax lockdown measures [2]. A clear decision making framework should guide these vitally important national/regional decisions.

In April 2020 the World Health Organization (WHO) issued a checklist of six lockdown exit criteria for countries to consider (**Box 1**) [3]. Our analysis has however found that many (particularly high-income) countries have chosen to formulate their own criteria, which do not always align with the WHO criteria (**Table 1**). These divergent approaches may reflect the limited traction that WHO has in many high-income countries, the speed and form of the initial response, and as a result, very different rates of infection. They may also reflect different stages of the pandemic that countries find themselves in and available health and other resources to respond to the challenges thrown up by COVID-19 [21,22].

Box 1 **WHO lockdown exit criteria.**

1. COVID-19 transmission is **controlled** to a level of sporadic cases and clusters of cases, all from known contacts or importations; at a minimum, new cases would be reduced to a level that the health system can manage based on **health care capacity**.

2. Sufficient public health workforce and health system **capacities** are in place to enable the major shift from detecting and treating mainly serious cases **to detecting and isolating all cases**, irrespective of severity and whether there is local transmission or an importation.

3. Outbreak **risks in high-vulnerability settings are minimised**, which requires all major drivers or amplifiers of COVID-19 transmission to have been identified, with appropriate measures in place to **maximise physical distancing and minimise the risk of new outbreaks**.

4. **Preventive measures** are established **in workplaces**.

5. **Manage the risk of exporting and importing cases** from communities with high risks of transmission.

6. **Communities are fully engaged** and understand that the transition away from large-scale movement restrictions and public health and social measures – from detecting and treating serious cases to detecting and isolating all cases – is a ‘new normal’ in which prevention measures would be maintained, and that all people have key roles in preventing a resurgence in case numbers.

**Table 1:** International lockdown exit criteria mapped to WHO recommendations

WHO recommendation	Recommendation 1: Controlling community transmission	Recommendation 2: Healthcare capacity	Recommendation 2: Detecting and isolating cases	Recommendation 3: Minimise outbreaks in high vulnerability settings	Recommendation 3: Maximise physical distancing and minimise the risk of new outbreaks
Austria [4]	×				
Czech Republic [5]	×				
Denmark [6]	×				
France [7,8]	×				
Ireland [9]	×	×	×	×	
Scotland [10]	×	×	×		×
Spain [11]	×	×			×
Italy [12]	×				
Belgium [13]	×				
United Kingdom [14]	×	×	×		
Germany [15]	×	×			×
Singapore [16]	×				
New Zealand [17,18]	×	×	×		×
Australia [17]		×			
United States [19]	×	×	×		
Canada [20]	×	×	×		

WHO – World Health Organization

Our analysis has identified areas of broad agreement in relation to controlling community transmission, adequate health care capacity, and detecting and isolating cases (WHO Recommendations 1 and 2, **Table 1**). The most common measurement criterion used internationally is the reduction of cases, but there is a lack of agreement of what precisely this reduction should be and countries have as a result developed different indicators. Examples include “new coronavirus infections stabilise at a low level” (Germany), “infection rates decreasing to manageable levels” (United Kingdom), or “sustained reduction in cases” (Canada). An exception is Singapore, which clearly states “bring down daily infections more sharply, to single digit, or even zero”. The most commonly used measurement is a reduction in reproduction rate, R_t_, to below 1.

Areas of divergence mainly relate to WHO Recommendations 3-6, concerning preventative measures for curbing spread in vulnerable communities and in workplaces, and the need for sustained community engagement. The lack of attention to prioritising highly vulnerable communities is of concern given that, for example, almost half of COVID-19 deaths in Europe have occurred in care homes [23].

Recommendation 5, tackling preventative measures in workplaces, is not mentioned in any lockdown exit criteria. This seems to be despite major concerns in the community. For example, Scotland’s Health and Safety Executive has already received 390 public concerns regarding workplace safety since March 2020, prior to any easing of lockdown measures [24].

Of note is also that, currently no country has included WHO Recommendation 6 in their lockdown exit criteria, aiming to ensure that communities understand public health prevention measures and take them seriously as they move to collectively contain transmission and mitigate the effects of the pandemic.

These medium- to long-term deliberations are crucial to ensuring the sustainability of managing the pandemic and should therefore be considered necessary conditions for exiting lockdown. These factors have an impact on community transmission and therefore play an important role in influencing overall success of strategies. For example, in order to effectively implement test, trace, and isolate measures, the public will need to understand that they may have to share some of their personal data to allow adequate contact tracing, an issue some – particularly those living in Western liberal democracies – may perceive as an infringement of civil liberties. Similarly, to keep the transmission rate low, the public will need to continue to implement and respect measures surrounding physical distancing in all settings including the workplace and social gatherings.

**Figure:**
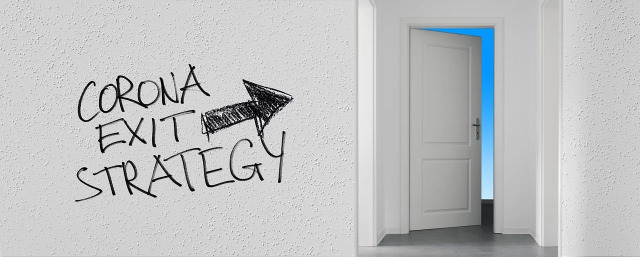
Photo: https://cdn.pixabay.com/photo/2020/04/14/05/59/door-5041047_1280.jpg.

Overall, our analysis shows that as countries move from the containment phase to longer-term thinking, there is a need to place greater emphasis on meaningful dialogue with the public to co-formulate sustainable engagement strategies and to avoid confusion and non-compliance to lockdown easing [25,26].

Our analysis will, we hope, allow countries to take stock of the range of approaches that they have employed thus far and help to refine their lockdown exit criteria by incorporating the need for genuine community dialogue and engagement. This will be imperative if countries are to develop a shared understanding of the ‘new normal’. It is hoped that early experiences and lessons will also be proactively shared between member nations through the WHO [27].
